# Defects in the Palatine Organs

**Published:** 1874-09

**Authors:** M. Bissell


### DEFECTS IN THE PALATINE ORGANS.

BY M. BISSELL.

Cases of defects in the palatine organs are not of very common occurrence in the practice of the dentist, but every operator who has been ten to fifteen years in practice may have met with one or more, either congenital, or caused by disease or from accident—and the defect may include the lip, the alveolar ridge, and embrace those important organs, the velum and uvula. From my observation I think the simple fissure in the palate process is more frequent than complicated cases.

When the velum and uvula are involved in the defect very careful manipulation is requisite to restore the parts by an operation to what the normal condition would have been, had not nature deviated from her usual course, or had not disease or accident interfered to abridge the comfort of the subject, and often, to a very great degree, prevent articulate sounds. If there is only a simple fissure in the palate process, or if the teeth are included, an obturator with teeth attached, is of comparatively easy construction, and such appliances will be found of very esssential service in restoring to a greater or less extent, the natural tones of the voice, and in preventing the liquids and solids of the food from passing into the nasal cavity and as speech consists of combinations of sounds produced by organs between the glottis and the external opening of the mouth, the co-operation of the several parts of the mouth are necessary for the formation of sounds; if one of these are defective or wanting the ability to produce sounds that are characteristic of humanity, is wholly or partially destroyed.

The practice of the dentist has enabled the sufferers from these lesions to speak with nearly their natural tones, whether the condition was caused from disease or accident or from congenital mal-formation. An interesting case of the restoration, artificially, of the loss, (by ruffian hands) of the velum and uvula, was delineated on the black board, at the meeting of the South Carolina State Dental Association, 1873. The appliance exhibited was beautifully executed by the operator, Dr. J. B. Patrick, of Charleston. The speech and of course the vocal powers of the subject was vastly interfered with, but after a reasonable use of the fixture, the patient stated that she could sing with her former facility. This is but one of several cases that have fallen into the hands of Dr. P., all of them have been successfully treated. This branch of practice is nearly a speciality with some of the profession north. In my own practice I have had but two cases of congenital fissure in the palate organs, where my services were required to close by an obturator the opening. In neither case were the velum or uvula involved and the teeth were perfect. As an obturator, a simple gold plate was fitted to the arch, supported by clasps extending along the palatine surfaces of the molars and resting in the buccal surfaces of the third molars the appliance answered the intended purpose—affording comfort to the patient and a natural tone of voice. I had expected to have a third and a more complicated case, in which my services would have been required, but have been disapointed by the party removing to a distant state.

The case referred to was the eldest of three children of one family, all of whom are afflicted with the abnormal condition of congenital fissure, in connection with loss of velum and cleft palate. The subjects are at this period aged fourteen, eleven and seven years. No doubt an operation on these subjects should have been performed, and I urged the father to submit their cases to one of the surgeons of the vicinity. My efforts would have been to construct an artificial appliance but I have not been successful in either respect, owing to the timidity of both mother and child, and to their removal.

The circumstances attending these cases as far I have learned them are, that the first subject of the abnormal condition was born in the early years of the late war. One evening when the mother "was, as ladies wish to be who love their lords" a stranger called at the house and desired to remain the night. His first utterance had a very unpleasant effect on the lady, as he was afflicted with a mal-formation of the palatine organs and the lady remarked to her husband that she feared the consequences of the stranger's visit and of his remaining; but the claims of hospitality outweighed the fears and he remained. The event would seem to prove that the seeds of injury were then sown, for in the fullness of time the child was born, and the mother's worst fears were realized. The lesion in this case embraced the palate process, the loss of velum and a cleft uvula. The fissure in this case was more extensive than the largest given in Harris' work. The cause of the defective formation would appear evident on the hypothesis, that impressions made on the mind of the mother, may be communicated to and impressed upon the fœtus although the doctrine is denied by many writers.

What appears extremely singular and interesting in connection with this case is the fact that the lady should have given birth, at her subsequent labor, to a child with almost an identical lesion of the parts, while the interest is not lessened from the additional fact of a child being born at her third pregnancy with nearly the same mal-formation. The first case presented a broad fissure in the palate process, with loss of velum, and uvula entirely devided, and presenting the appearance of two distinct uvuli. The second case presented a fissure less in width, about the same in length as the first, loss of velum and cleft uvula; but with this feature not as distinctly marked as in the first case. The condition of the organs in the third child is, the loss of the velum, the fissure in the arch, but less in length and width, a uvula of very small size, and but slightly slit on one side. The fissures in each case involved the median line, but neither of them included the upper lip, or the alveolar ridge.

It might possibly have been of some interest to have ascertained the period of gestation when the first impression was made on the mother's mind, perhaps during the formation of the glottis and its appendages. Were the impressions so firmly stamped upon the brain, in the first instance, as to withstand the eroding effects of time, and probably in connection with the defective speech of the first child, aid in producing a second mal-formation? Did the defective formation in the first and second aid, with the first unfortunate impression, in impressing the abnormal condition on the third child?

These questions may be entirely pertinent, if the hypothesis is entitled to credit, but just here the theory may loom up that there is no nervous connection between the brain of the mother and the embryo being to produce such impressions, a doctrine held by Tyler Smith and other writers. That doctrine admitted and any fine spun theories of impressions must fail.

What then was the cause of this abnormal condition in the three children, if the "marking" can not be accounted for on the theory of impressions made on the mother's mind during pregnancy? What of the statement made by Draper, that the offspring of a second marriage on the part of a woman, may have some of the lineaments of the first husband impressed upon it. If this is true, the peculiarity must have had a local habitation somewhere in the female, if not in the brain, was it impressed on the creative functions within the pelvic organs, and from thence imparted to the fœtus?

The recent case of Jesse Pomeroy, the child murderer and mutilator, of Boston, appears to be an instance of the impressions made through the mind of the mother on the fœtus. The mother, while pregnant with this child, took much pleasure in witnessing her husband, (who was a butcher,) kill sheep and cattle, and she often assisted him, for the mere pleasure of seeing the animals killed. The boy, as soon as he could handle a knife, indulged himself in sticking the knife into pieces of meat, and continued the practice as he grew up, and assisted his father, increasing the propensity for blood, which it would seem may have been impressed upon the brain while in embryo by his mother's indulgence. He was "marked" by the mother, not externally, but internally, and effectually, judging from the number of victums he operated upon. Must this case be considered hereditary, as is asserted for cases of mal-formations. It is not probable that the child when he first amused himself with the knife and meat, had so frequently witnessed butchering by his father, as to have acquired a decided taste for the amusement, and the sight of blood, had not some unusual impression been made on his brain, and he "did it, because he could not help doing it," as he told the judge.

Are the lesions to be regarded as hereditary peculiarities as is asserted for some abnormal formations, for instance, six fingers on the hands, and six toes on the feet, from generation to generation. In cases of hereditary transmission, how is the malformation impressed upon the fœtus? Has the germ of the uncreated being the identical malformations? If not, and the mother can not impress them through her nervous system from whence comes this hereditary diathesis?

In reference to the foregoing cases of congenital fissure, on enquiry, there could not be traced any hereditary tendency to mal-formations on the part of either parent, or the grandparents for two generations at least.

It would be interesting in this connection, to ascertain whether the mother has borne any children since the third case referred to, with corresponding mal-formations. Possibly not, as the cases reported had "grown small by degrees, and beautifully less," in extent of mal-formation, the proclivity may may have died out. In my suggestions, in connection with the foregoing statements, I have no doubt trenched upon sacred ground, and should have "removed the shoes from off my feet," before entering the precincts, but it is not the first, and probably it will not be the last instance of fools rushing in, where angels dared not tread.

